# Safety evaluation of aqueous extracts of *Sanghuangporus vaninii* fruiting body in Sprague–Dawley rats

**DOI:** 10.1002/fsn3.1811

**Published:** 2020-08-05

**Authors:** Jinxi Huo, Yuqing Sun, Shi Zhong, Yougui Li, Ruchun Yang, Lijuan Xia, Jiahong Wang, Majuan Zhang, Jianxun Zhu

**Affiliations:** ^1^ Sericultural Research Institute Zhejiang Academy of Agricultural Sciences Hangzhou China; ^2^ Hangzhou Hospital of Traditional Chinese Medicine Hangzhou China; ^3^ Center of Safety Evaluation Zhejiang Academy of Medical Sciences Hangzhou China

**Keywords:** acute toxicity, aqueous extracts, repeated dose toxicity, safety evaluation, *Sanghuangporus vaninii*

## Abstract

*Sanghuangporus vaninii*, called “Sanghuang,” is orally used for health care, tumor, and inflammation treatment in Asia. However, the safety of *S. vaninii* has not been evaluated. The major compounds analysis showed that aqueous extracts of *S. vaninii* fruiting body were rich in polysaccharides, nucleotides, and polyphenols. Then, the aqueous was given orally to Sprague–Dawley rats for toxical test. In acute toxicity study, the maximum tolerated dose was 21 g/kg. In 17‐week repeated dose toxicity experiment, all rats had no abnormal reaction among control group, low dose group (0.15 g/kg) and middle dose group (1.00 g/kg). At high dose group (6.00 g/kg), the feces began to darken on 16th day (D16), and turned to drug stained stool on D21, all rats recovered on the 3rd day (D92) of recovery period. During the whole experiment, there were no animal death and no treatment‐related changes in any of the parameters under the all doses. These results indicated the No‐Observed Adverse Effect Level of aqueous extract of *S. vaninii* fruiting body was 1.0 g/kg.

## Graphical abstract

The LD 0 and NOAEL of aqueous extracts of *Sanghuangporus vaninii* fruiting body were 21 and 1.0 g/kg, respectively. These data suggested *S. vaninii* could be considered as nonpathogenic and safe for human consumption as a traditional Chinese medicine for daily health preservation.

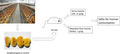

## 1 INTRODUCTION

The genus *Sanghuangporus* (e.g., *baumii*, *linteus*, *gilvus*, and *vaninii*), also called “Sanghuang” in China, are famous medicinal mushroom (Lee, Cho, Kim, Hong, & Yoo, 1996; Zhu, Song, Zhou, Si, & Cui, 2019). Sanghuang have been used in Chinese traditional medicine for over 2000 years to treat various diseases. In recent years, modern pharmacological researches display that Sanghuang have multifunction such as anti‐carcinogenesis, anti‐inflammatory, anti‐oxidative, anti‐fungal, and immunomodulatory activities (Cai et al., 2019; Chen et al., 2019; Huo et al., 2020; Zhu & Cui, 2016). These active effects provided great potential for their application in medicinal and food industries.

Although no evidence suggested Sanghuang had toxic effects, and the safety of *S. linteus* mycelia had been evaluated (Li, Chen, Sheu, Huang, & Chen, 2020). Some researches showed the fruiting body had higher medicinal effect (Huo et al., 2020; Shao et al., 2019). However, the safety of Sanghuang fruiting body had not been evaluated. *Sanghuangporus vaninii* is a widely used and valuable strain in China, in the present study, aqueous extracts of *S. vaninii* fruiting body was given orally to Sprague–Dawley (SD) rats for acute and repeated dose toxicity experiment. The results suggested that there was no obvious toxic target organ of *S. vaninii*, which supported its safety for human consumption.

## 2 MATERIAL AND METHODS

### 2.1 Test sample preparation

The fruiting body of *S. vaninii* was cultivated at Sericultural Research Institute, Zhejiang Academy of Agricultural Sciences. The cultivated medium included mulberry sawdust 50%, cottonseed hull 28%, bran 20%, sugar 1%, and gypsum 1%. The inoculated bags were cultured at 25°C and 80% humidity for 5–6 months for harvesting mature fruiting body. The mature fruit body was sliced and dried at 50°C for 48 hr by oven. The dried fruiting body of *S. vaninii* was extracted with boiling water (w/v = 1:10) for 2 hr, the filtrated aqueous extracts were concentrated by rotary evaporation and lyophilized by freeze vacuum drying to obtain aqueous extracts powders. The dried powders were stored at −20°C for use.

### 2.2 Major compounds analysis

The total content of polysaccharides was analysis according to phenol–sulfuric acid method (Masuko et al., 2005). The total content of polyphenols was measured using Folin‐Ciocalteu method (Ma et al., 2019). The major compounds were identified by high‐performance liquid chromatography (HPLC) and high resolution electrospray ionization mass spectroscopy (HR‐ESI‐MS) method (Huo et al., 2020).The aqueous extracts treated with three volumes of ethanol, the supernatant was collected after centrifugation for HPLC and HR‐ESI‐MS analysis.

### 2.3 Test animals

Sprague–Dawley rats were purchased from Shanghai SLAC Laboratory Animal Co., Ltd, animal certification number was 2015000546233 (male rats, 22), 2015000546233 (female rats, 22), 2015000546235 (male rats, 65), and 2015000546236 (female rats, 65). The rats were 5–7 weeks old with a mass from 132 to 165 g of SPF grade. Male and female rats were fed in separate cages. During the period of administration, each cage had five rats, and during the observation period of recovery period, each cage had 2–3 rats.

### 2.4 Toxicity studies

Prothrombin time (PT) test kit, thrombin time (TT) test kit, hematology quality control, reticulocyte (RETIC) reagent, rinse sheath, complete blood count (CBC) timepac kit, differential blood count (DIFF)Timepac kit were purchased from Siemens Healthcare Diagnostics. Alanine aminotransferase (ALT) test kit, Aspartate aminotransferase (AST) test kit, Albumin (ALB) test kit, Alkaline phosphatase (ALP) test kit, Glucose (GLU) test kit, Total protein test (T. P) Kit, Total bilirubin (T. BIL) test kit, Total cholesterol (T. CHO) test kit, Urea (BUN) test kit, γ‐Glutamyltransferase (γ‐GT) test kit, Creatinine (Crea) liquid test kit, Creatine kinase (CK) test kit, Triglyceride (TG) test kit were purchased from DiaSys Diagnostic Systems GmbH. Urinalysis strip was purchased from Changchun Dirui Medical Technology Co., Ltd. Compound Tropicamide Eye Drops were purchased from Shenyang Xingqi ophthalmic Co., Ltd.

#### 2.4.1 Acute toxicity study

The volume of single oral gavage was 3.0 ml/100 g body weight, and the interval of administration was 6–7 hr. Two times a day, the total dose volume was 6.0 ml/100 g (21, 24, and 27 g/kg) body weight. After administration, clinical signs were observed in treated animals at 0.5, 1, 2, 4, 5, 6, 7, 8 hr, and daily once thereafter for 15 consecutive days. Cage side observations include changes appearance, behavior, mental state, appetite, urine, stool, secretions, and death of rats. Animals were euthanized after 15 days of observations.

#### 2.4.2 17‐Week repeated dose toxicity study

**FIGURE 1:**
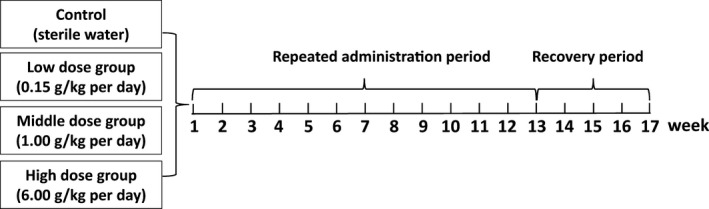
Model of 17‐week repeated dose toxicity study

The study was carried out to evaluate the toxicity of the test item after 13 weeks of repeated administration through oral route and 4 weeks recovery in rats, for an indication of the dose–response relationship and the determination of the No‐Observed Adverse Effect Level (NOAEL) (Figure 1). The low dose group was set as 0.15 g/kg per day, the middle dose group was set as 1.00 g/kg per day and the high dose group as 6.00 g/kg per day. The control was sterile water. The volume of daily oral gavage was 1.5 ml/100 g body weight, and the oral gavage administration lasted for 13 weeks.

#### 2.4.3 General observation

Observation of rats' appearance and physical signs included skin, mouth, nose, behavior, respiration, gland secretion, fecal, and urine characteristics. During the administration period, the general observation was carried out before and after the administration in the morning every day. The degree and recovery time of symptoms were observed, and the gender difference and frequency were also observed. The general observation during the recovery period was carried out in the morning every day.

#### 2.4.4 Body weight

The weight of rats was recorded before repeated toxicity test. Then, the weight was recorded once a week during test.

#### 2.4.5 Feed consumption

The feed consumption was measured once a week during the administration period and the recovery period of drug withdrawal. The feeding amount on the first day was measured, and the remaining amount in the same period of the next day was subtracted as the feeding amount of each cage. Then, the average feeding amount of each cage per day was calculated by dividing the actual number of animals on each cage.

#### 2.4.6 Hematology, clinical biochemistry, and urine analysis

Hematology was measured in the examination of drug withdrawal (13 weeks of administration) and the examination of the end of recovery period (4 weeks of drug withdrawal). Before blood collection, the rats were fasted for more than 12 hr and were injected 50 mg/kg pentobarbital sodium intraperitoneally for anesthesia, and blood was collected from the abdominal aorta.

#### 2.4.7 Ophthalmic examination

Compound tropicamide eye drops are used for mydriasis (1 drop/eye). The eyelid, orbit, lacrimal apparatus, conjunctiva, cornea, and sclera were examined with naked eye in bright light, and then the fundus was examined with ophthalmoscope.

#### 2.4.8 Gross and histopathological examination

Pathological section examinations of rats were at drug withdrawal and the end of recovery period. Rats were dissected before fasting for at least 12 hr. According to the body weight, rats were anesthetized with pentobarbital sodium (intraperitoneal injection of 50 mg/kg dose). Organs were weighed and histopathologically examined.

### 2.5 Statistical analysis

Data were described by mean ± standard deviation, and one‐way ANOVA was used for significance analysis. Three groups (low, middle, and high) were compared with the control group, * represented *p* < .05, ** represented *p* < .01.

## 3 RESULTS

### 3.1 Aqueous extracts of *S. vaninii* fruiting body was rich in polysaccharides and polyphenols

**FIGURE 2:**
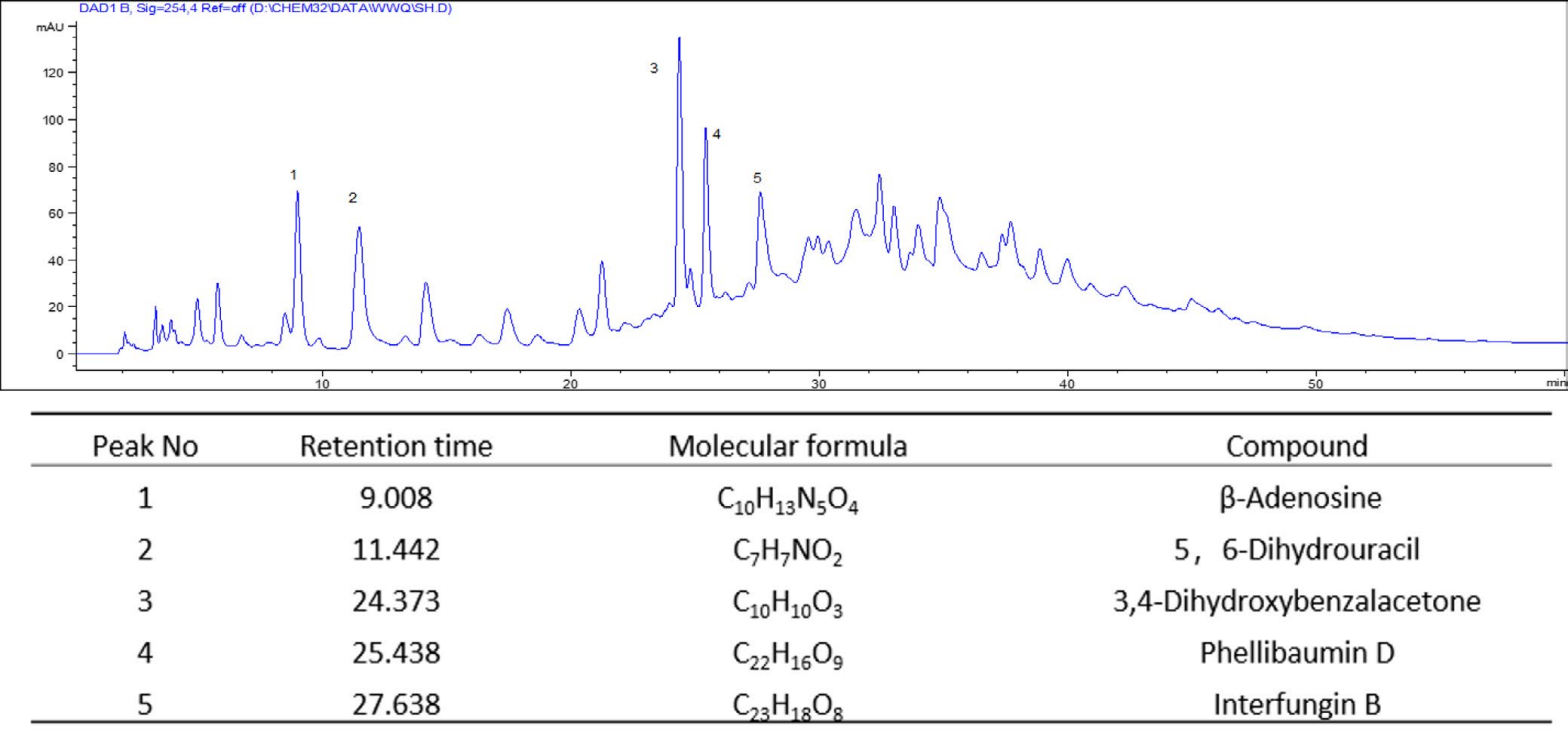
Major compounds analysis of aqueous extracts of *S. vaninii* fruiting body

Previous studies have found that Sanghuang were mainly rich in polysaccharides, triterpenoids, and polyphenols. The content of total polysaccharides and polyphenols were 5.51% and 23.00% according to phenol–sulfuric acid method and Folin‐Ciocalteu method, respectively. In order to further identify the components of bioactive small molecules in the aqueous extracts, we conducted HPLC and HR‐ESI‐MS analysis. As the Figure 2 shown, the main peaks are nucleotides and polyphenols (Figure S1).

### 3.2 Acute toxicity study

**FIGURE 3:**
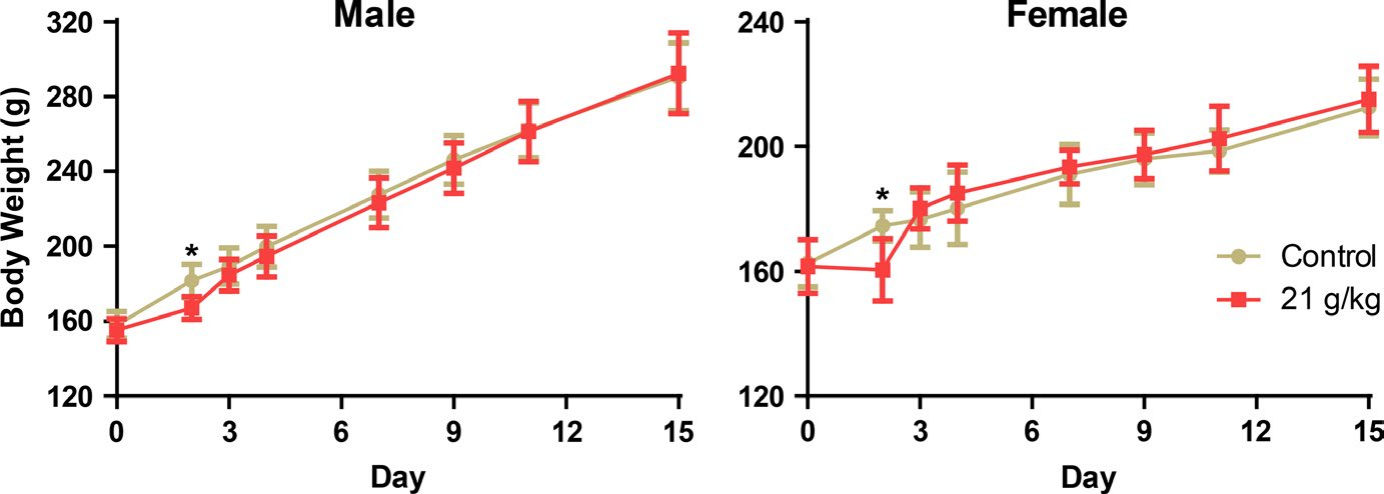
The weight of rats in acute study

Under the dose of 24 and 27 g/kg of aqueous extracts, there was death and the mortality rate of rats was 30% and 80%, respectively. Under the dose of 21 g/kg of aqueous extracts, the main toxic reactions of the rats showed activity reduction, prone, loose stool, perianal filth, tremor at rest. On the fourth day, all the experimental rats returned to normal. The aqueous extracts had a short‐term reversible effect on the weight growth of rats. The weight of male and female rats was lighter than that of the control group on 2nd day, the body weight of male and female rats increased with the prolongation of the test time, the same as the control group (Figure 3). There no mortality was observed during the experiment.

### 3.3 17‐Week repeated dose toxicity study

#### 3.3.1 General observation

During the whole experiment, all rats in the control group, low dose group (0.15 g/kg), and middle dose group (1.00 g/kg) had no abnormal reaction. At the dose of 6.00 g/kg (high dose group), the feces of all rats began to darken on the 16th day (D16), and turned to drug stained stool on the D21, and recovered on the 3rd day (D92) of recovery period. The male and female rats appeared slight salivation immediately for 15 min after administration from D24 and D36, respectively. The salivation rate of male and female rats was 100%. During the recovery period of drug withdrawal, the above reactions recovered quickly. There no mortality was observed during the experiment.

#### 3.3.2 Body weight

**FIGURE 4:**
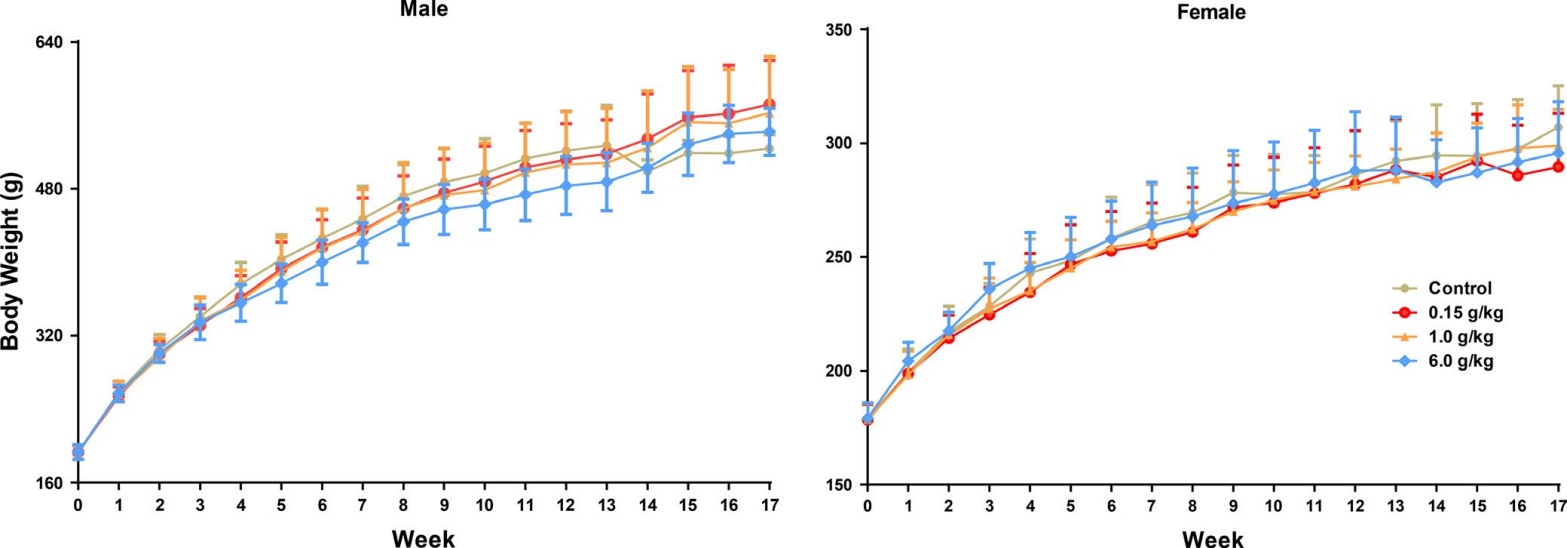
The weight of rats in 17‐week repeated dose toxicity study

During the whole experiment, the dosage of 0.15, 1.00, and 6.00 g/kg of aqueous extracts had no significant effect on the weight growth of female rats (Figure 4).The dosage of 6.00 g/kg of aqueous extracts could slow down the weight growth of male rats during the administration period, the body weight growth of the male rats in the high dose group was slower than that of the control group in the corresponding period from the 5th week (W5) to the 13th week (W13) of administration; however, there was no statistical difference between the body weight of the male rats in each dose group (Figure 4).

#### 3.3.3 Feed consumption

**FIGURE 5:**
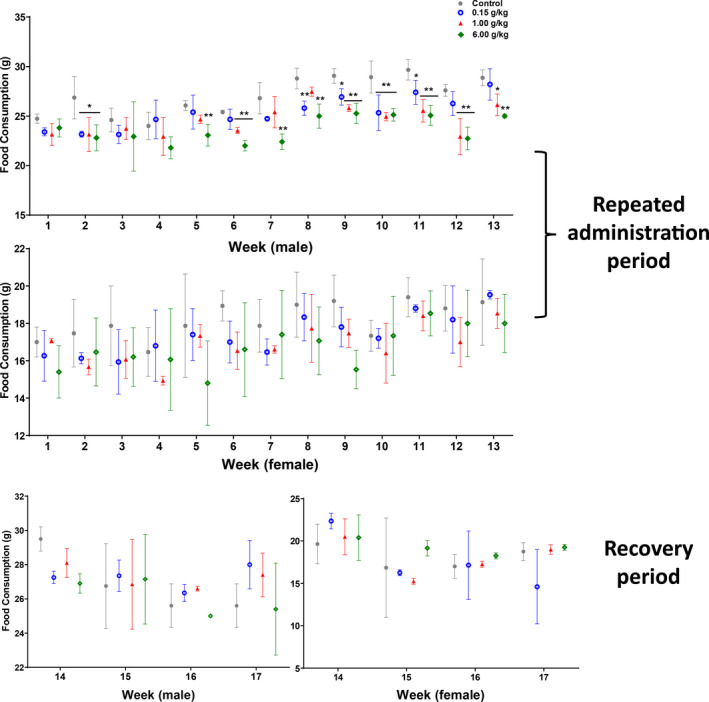
The food consumption of rats in 17‐week repeated dose toxicity study

The aqueous extracts (0.15, 1.00, 6.00 g/kg) had the effect of reducing the food consumption of male rats, but had no effect on the food consumption of female rats (Figure 5). In the low dose group, the consumption of male rats decreased at W2 and W8‐W11, in the middle dose group, at W2, W6, W9‐W13, and in the high dose group, at W2 and W5‐W13. In addition, the food consumption of 6.00 g/kg in female rats decreased in a short time at the W9 of administration; however, the decrease was slight, and only occurred at a single time point, considering no toxicological significance (Figure 5).

#### 3.3.4 Hematology, clinical biochemistry, and urine analysis

The dosage of 0.15, 1.00, and 6.00 g/kg of aqueous extracts had no effect on the hematological indexes of rats at the end of drug withdrawal and recovery period (Tables S1 and S2). At the end of drug withdrawal, HGB and HCT decreased in low dose group. RBC, HGB, and HCT decreased, MPV increased in middle dose group. HGB, HCT, and RDW decreased and MPV increased in high dose group (Table S1). At the end of the recovery period, PLT increased in the low, middle, and high dose groups, while EOS and LUC increased in the middle dose group (Table S2).

There were no abnormal effects of low, middle, and high dose groups on most of serum biochemical indexes of rats during drug withdrawal and recovery period (Tables S3 and S4). At the end of drug withdrawal, GLU decreased in the low, middle, and high dose group, Na^+^ increased in the low dose group, Na^+^, Cl^‐^ increased and TCa decreased in the middle and high dose groups, and T.BIL increased in the high dose group (Table S3). At the end of recovery period, TCa and GLO decreased in low, middle, and high dose groups (Table S4).

The middle and high dosages of aqueous extracts had an effect on the urine index of male rats after drug withdrawal. At the middle dosage, the specific gravity increased, and at the high dosage, the specific gravity increased and pH decreased (Table S5). There were no abnormal effects on female rats (Table S6). There were no abnormal effects on serum biochemistry indexes of male rats in all groups at the recovery period (Table S7). The microalbumin of female rats increased at the end of the recovery period in high dosage group (Table S8).

#### 3.3.5 Ophthalmic examination

There were no effects on the ophthalmic indexes of rats in all dosages groups (Table S9).

#### 3.3.6 Organs weight and organs coefficient

There was no significant abnormal effect of all dosages groups on organ weight and organ coefficient at the end of drug withdrawal and recovery period (Tables S10–S13). The liver weight of the male rats in the low, middle, and high dose groups decreased, the kidney coefficient of the high dose group increased at the end of drug withdrawal (Table S10). The paranephros weight of male rats in the low‐dose group was increased at the end of the recovery period with a small change range (Table S12).

#### 3.3.7 Gross and histopathological examination

No test‐related gross and toxic pathological changes were found at the end of drug withdrawal examination and recovery period examination. No gross pathological changes were found in the cut‐off examination. At the end of drug withdrawal examination and recovery period examination, it can be seen that the pathological changes of lung, liver, thyroid, bladder, and other organs in individual rats, and there is no dose correlation, which suggested that there no toxicological significance.

## 4 DISCUSSION

*Sanghuangporus vaninii*, a species of Sanghuang, is a traditional medicinal fungus. The fruiting body of *S. vaninii* can be used as a traditional Chinese medicine for daily health preservation and adjuvant therapy (Chen et al., 2019). However, its safety had not been assessed. In the present study, the acute and repeated dose toxicities of aqueous extracts of *S. vaninii* were assessed in SD rats based on Good Laboratory Practice (GLP, China, 2017).

Chen et al. (2019) found that Sanghuang were mainly rich in polysaccharides, triterpenoids, and polyphenols. We found the aqueous extracts of *S. vaninii* fruiting body were rich in polysaccharides, nucleotides, and polyphenols (Figure 2), these polyphenols were representative compounds in Sanghuang (Chen et al., 2019).

Acute toxicity testing is a fundamental test for assessing safety (Donohue & Salminen, 1996) and has been applied in various safety assessment studies (Strickland et al., 2020). In the acute toxicity study, the maximum tolerated dose (LD 0) was 21 g/kg.

Because *S. vaninii* was usually used for daily health preservation, it is necessary to assess repeated dose toxicity. In the 17‐week repeated dose toxicity study, three doses were assessed on SD rats, the low‐dose group is equivalent to six times of the recommended clinical dose of human, the high dose group was set as 6.00 g/kg (equivalent to 240 times of the clinical dosage).

After administration of 6.00 g/kg, the stool color deepened, and there was no abnormal reaction at middle and low doses. There was a certain dose‐time relationship. It was considered that the high concentration and large volume of aqueous extracts were rich in pigment or flavonoids, which were not completely absorbed.

Although the aqueous extracts had the effect of reducing the food consumption of male rats (Figure 5), the body weight of male rats in each dose group was not statistically different from that in control group during the whole experiment period. But combined with the specific data of the body weight of male rats, the body weight growth of male rats in the high dose group was slower than the control group from the 5th week to the 13th week of administration (Figure 4), considering that the high dose group could slow down the body weight growth of male rats during the administration period. According to the statistical results of body weight and food consumption, there is a certain dose time relationship, suggesting that the high concentration and large volume of the aqueous extracts might reduce appetite of male rats.

At the end of drug withdrawal, HGB, HCT, RBC, RDW, and MPV in each dose group were abnormal individually, but the sensitive indexes of hematopoiesis of bone marrow erythrocytes and RETIC were normal (Table S1), and the histopathological results showed that there was no abnormal change of hematopoiesis of bone marrow, which suggested there no obvious toxicological significance. At the end of the recovery period, PLT increased in some dose group, while other indexes of platelets and coagulation indexes had no obvious abnormality (Table S2), suggesting that the statistical difference might be caused by the fluctuation of the test value.

In the urine analysis, some rats in each group were abnormal including microalbumin, ketone body, protein, leukocyte, urobilinogen, and nitrite (Table S5). However, according to the pathological results, there were no abnormal pathological changes in the kidney and liver, suggesting it was the fluctuation of urine test, there was no significant toxicological significance.

Above all, the NOAEL was 1.0 g/kg (the middle dose group). The aqueous extracts of *S. vaninii* could be used for daily health care by oral administration under clinical dose safely.

## 5 CONCLUSION

Based on the observations and data, the LD 0 of aqueous extracts of *S. vaninii* fruiting body was 21 g/kg and NOAEL was 1.0 g/kg. There were no obvious toxic target organs were found. Therefore, *S. vaninii* could be considered as nonpathogenic and safe for human consumption as a traditional Chinese medicine for daily health preservation.

## CONFLICT OF INTEREST

The authors declare that they have no conflict of interests.

## ETHICAL APPROVAL

All the animal experiments were carried out at Center of Safety Evaluation, Zhejiang Academy of Medical Sciences. The organization has been certificated by AAALAC (Association for assessment and Accreditation of Laboratory animal care, International Commission for the assessment and management of laboratory animals). The use of laboratory animals has been approved by the Department of Science and Technology of Zhejiang Province, and the license number of experimental animal is SYXK (Zhejiang) 2017‐0010. This study was carried out in strict accordance with the recommendations in the Guide for the Care and Use of Laboratory Animals of the National Institutes of Health. The protocol was approved by IACUC (Institutional Animal Care and Use Committee) of Zhejiang Academy of Medical Sciences (Protocol Number: 17018CD1). All surgery was performed under sodium pentobarbital anesthesia, and all efforts were made to minimize suffering.

## Supporting information

Supplementary Material
