# Supplementary material for: Safety evaluation of aqueous extracts of Sanghuangporus vaninii fruiting body in Sprague–Dawley rats
Source: Food Sci Nutr. 2020 Aug 5;8(9):5107–13. doi: 10.1002/fsn3.1811 (PMC7500762; doi:10.1002/fsn3.1811)
Supplement: Supplementary file 1 — Supplementary Material [file FSN3-8-5107-s001.docx]

**Supporting materials**

**Table S1 Effect of aqueous extracts on hematological indexes in rats after drug withdrawal**

| Test items | Groups | | | | | | | | | | | |  |
| --- | --- | --- | --- | --- | --- | --- | --- | --- | --- | --- | --- | --- | --- |
|  | Control | | | Low dose group | | | Middle dose group | | | High dose group | | | |
| Number of rats | 20 | | | 20 | | | 20 | | | 20 | | |  |
| WBC（10^3^/μL） | 5.02 | ± | 1.95 | 5.25 | ± | 2.40 | 4.44 | ± | 1.80 | 4.67 | ± | 1.62 |  |
| RBC（10^6^/μL） | 8.22 | ± | 0.59 | 7.94 | ± | 0.58 | 7.69 | ± | 0.65** | 7.91 | ± | 0.53 |  |
| HGB（g/dL） | 147 | ± | 9 | 142 | ± | 6* | 138 | ± | 8** | 142 | ± | 8* |  |
| HCT（%） | 46.1 | ± | 3.0 | 44.4 | ± | 2.3* | 42.9 | ± | 2.7** | 44.0 | ± | 2.4* |  |
| MCV（fL） | 56.1 | ± | 1.9 | 56.0 | ± | 1.9 | 56.0 | ± | 2.0 | 55.7 | ± | 1.7 |  |
| MCH（pg） | 17.9 | ± | 0.8 | 17.9 | ± | 0.8 | 18.0 | ± | 0.8 | 17.9 | ± | 0.7 |  |
| MCHC（g/L） | 319 | ± | 10 | 319 | ± | 8 | 321 | ± | 9 | 322 | ± | 7 |  |
| RDW（%） | 12.1 | ± | 0.6 | 12.0 | ± | 0.7 | 12.2 | ± | 1.3 | 11.3 | ± | 0.4** |  |
| PLT（10^3^/μL） | 999 | ± | 104 | 991 | ± | 151 | 977 | ± | 132 | 977 | ± | 101 |  |
| MPV（fL） | 7.4 | ± | 0.7 | 7.5 | ± | 0.4 | 7.8 | ± | 0.5* | 8.0 | ± | 0.5** |  |
| %NEUT（%） | 14.6 | ± | 8.7 | 18.3 | ± | 10.4 | 19.6 | ± | 8.1 | 16.4 | ± | 9.6 |  |
| %LYMPH（%） | 79.9 | ± | 9.1 | 76.5 | ± | 10.5 | 74.7 | ± | 9.0 | 78.8 | ± | 9.6 |  |
| %MONO（%） | 2.5 | ± | 1.0 | 2.2 | ± | 0.8 | 2.6 | ± | 0.9 | 2.0 | ± | 0.7 |  |
| %EOS（%） | 2.1 | ± | 0.9 | 2.0 | ± | 0.7 | 2.1 | ± | 0.6 | 1.9 | ± | 0.4 |  |
| %BASO（%） | 0.1 | ± | 0.1 | 0.1 | ± | 0.1 | 0.1 | ± | 0.1 | 0.1 | ± | 0.1 |  |
| %LUC（%） | 0.8 | ± | 0.4 | 0.8 | ± | 0.3 | 0.9 | ± | 0.4 | 0.8 | ± | 0.3 |  |
| #NEUT（10^3^/μL） | 0.70 | ± | 0.45 | 0.90 | ± | 0.57 | 0.86 | ± | 0.49 | 0.78 | ± | 0.61 |  |
| #LYMPH（10^3^/μL） | 4.07 | ± | 1.80 | 4.08 | ± | 2.05 | 3.33 | ± | 1.41 | 3.67 | ± | 1.36 |  |
| #MONO（103/μL） | 0.11 | ± | 0.04 | 0.11 | ± | 0.07 | 0.12 | ± | 0.08 | 0.09 | ± | 0.04 |  |
| #EOS（10^3^/μL） | 0.11 | ± | 0.06 | 0.11 | ± | 0.06 | 0.09 | ± | 0.04 | 0.09 | ± | 0.04 |  |
| #BASO（10^3^/μL） | 0.01 | ± | 0.01 | 0.01 | ± | 0.01 | 0.01 | ± | 0.01 | 0.01 | ± | 0.01 |  |
| #LUC（10^3^/μL） | 0.04 | ± | 0.02 | 0.04 | ± | 0.03 | 0.04 | ± | 0.03 | 0.03 | ± | 0.01 |  |
| %RETIC（%） | 2.50 | ± | 0.50 | 2.36 | ± | 0.49 | 2.37 | ± | 0.46 | 2.31 | ± | 0.31 |  |
| #RETIC（10^9^/μL） | 203.7 | ± | 32.0 | 186.5 | ± | 34.8 | 180.8 | ± | 30.5 | 182.6 | ± | 25.3 |  |
| PT（s） | 8.3 | ± | 0.6 | 8.3 | ± | 0.5 | 8.2 | ± | 0.8 | 8.5 | ± | 0.6 |  |
| Fbg（g/L） | 1.850 | ± | 0.252 | 1.834 | ± | 0.227 | 1.970 | ± | 0.298 | 1.706 | ± | 0.242 |  |
| APTT（s） | 13.1 | ± | 2.0 | 13.7 | ± | 1.7 | 13.3 | ± | 1.8 | 14.2 | ± | 1.4 |  |
| TT（s） | 50.4 | ± | 11.3 | 56.4 | ± | 9.9 | 53.5 | ± | 13.1 | 58.1 | ± | 8.5 |  |

**Table S2 Effect of aqueous extracts on hematological indexes in rats at the end of recovery period**

| Test items | Groups | | | | | | | | | | | |
| --- | --- | --- | --- | --- | --- | --- | --- | --- | --- | --- | --- | --- |
|  | Control | | | Low dose group | | | Middle dose group | | | High dose group | | |
| Number of rats | 10 | | | 10 | | | 10 | | | 10 | | |
| WBC（10^3^/μL） | 4.19 | ± | 2.15 | 3.93 | ± | 1.66 | 5.14 | ± | 1.93 | 4.28 | ± | 1.23 |
| RBC（10^6^/μL） | 8.27 | ± | 0.60 | 8.22 | ± | 0.87 | 8.10 | ± | 0.59 | 8.30 | ± | 0.57 |
| HGB（g/dL） | 145 | ± | 9 | 145 | ± | 11 | 140 | ± | 4 | 145 | ± | 5 |
| HCT（%） | 45.6 | ± | 2.9 | 45.7 | ± | 4.3 | 44.3 | ± | 2.1 | 45.6 | ± | 1.6 |
| MCV（fL） | 55.2 | ± | 1.5 | 55.6 | ± | 1.6 | 54.9 | ± | 2.3 | 55.0 | ± | 2.2 |
| MCH（pg） | 17.6 | ± | 0.6 | 17.7 | ± | 0.9 | 17.4 | ± | 0.9 | 17.5 | ± | 0.8 |
| MCHC（g/L） | 318 | ± | 4 | 319 | ± | 8 | 317 | ± | 9 | 318 | ± | 7 |
| RDW（%） | 12.0 | ± | 0.6 | 11.5 | ± | 0.6 | 12.3 | ± | 2.3 | 11.7 | ± | 0.7 |
| PLT（10^3^/μL） | 919 | ± | 21 | 1121 | ± | 119** | 1085 | ± | 81** | 1046 | ± | 122* |
| MPV（fL） | 7.5 | ± | 0.5 | 7.4 | ± | 0.6 | 7.0 | ± | 0.4 | 7.1 | ± | 0.6 |
| %NEUT（%） | 17.7 | ± | 6.4 | 16.1 | ± | 4.6 | 16.8 | ± | 6.4 | 14.1 | ± | 5.6 |
| %LYMPH（%） | 77.2 | ± | 7.0 | 79.2 | ± | 4.9 | 77.7 | ± | 6.9 | 80.9 | ± | 5.7 |
| %MONO（%） | 2.5 | ± | 1.1 | 2.3 | ± | 0.7 | 2.4 | ± | 0.7 | 2.2 | ± | 0.6 |
| %EOS（%） | 2.0 | ± | 0.5 | 1.9 | ± | 0.5 | 2.2 | ± | 0.7 | 2.1 | ± | 0.4 |
| %BASO（%） | 0.1 | ± | 0.1 | 0.1 | ± | 0.1 | 0.1 | ± | 0.1 | 0.1 | ± | 0.1 |
| %LUC（%） | 0.6 | ± | 0.2 | 0.5 | ± | 0.2 | 0.7 | ± | 0.3 | 0.7 | ± | 0.2 |
| #NEUT（10^3^/μL） | 0.65 | ± | 0.26 | 0.66 | ± | 0.38 | 0.89 | ± | 0.51 | 0.61 | ± | 0.32 |
| #LYMPH（10^3^/μL） | 3.33 | ± | 1.83 | 3.09 | ± | 1.25 | 3.98 | ± | 1.49 | 3.46 | ± | 1.01 |
| #MONO（10^3^/μL） | 0.10 | ± | 0.07 | 0.09 | ± | 0.04 | 0.12 | ± | 0.05 | 0.09 | ± | 0.02 |
| #EOS（10^3^/μL） | 0.08 | ± | 0.03 | 0.07 | ± | 0.03 | 0.11 | ± | 0.05* | 0.08 | ± | 0.02 |
| #BASO（10^3^/μL） | 0.01 | ± | 0.01 | 0.00 | ± | 0.01 | 0.01 | ± | 0.01 | 0.01 | ± | 0.01 |
| #LUC（10^3^/μL） | 0.02 | ± | 0.02 | 0.02 | ± | 0.01 | 0.04 | ± | 0.01* | 0.03 | ± | 0.01 |
| %RETIC（%） | 2.26 | ± | 0.42 | 2.18 | ± | 0.29 | 2.11 | ± | 0.26 | 1.94 | ± | 0.27 |
| #RETIC（10e9/μL） | 186.0 | ± | 30.8 | 178.0 | ± | 23.3 | 171.3 | ± | 24.7 | 160.5 | ± | 19.2 |
| PT（s） | 8.3 | ± | 0.6 | 8.6 | ± | 1.0 | 8.2 | ± | 0.7 | 8.8 | ± | 0.9 |
| Fbg（g/L） | 1.787 | ± | 0.302 | 1.789 | ± | 0.458 | 1.592 | ± | 0.259 | 1.732 | ± | 0.394 |
| APTT（s） | 15.1 | ± | 1.5 | 14.8 | ± | 2.5 | 14.8 | ± | 1.9 | 16.2 | ± | 3.9 |
| TT（s） | 61.4 | ± | 4.7 | 54.3 | ± | 10.1 | 62.0 | ± | 5.5 | 54.7 | ± | 8.9 |

**Table S3 Effect of aqueous extracts on serum biochemistry in rats after drug withdrawal**

| Test items | Groups | | | | | | | | | | | |
| --- | --- | --- | --- | --- | --- | --- | --- | --- | --- | --- | --- | --- |
|  | Control | | | Low dose group | | | Middle dose group | | | High dose group | | |
| Number of rats | 20 | | | 20 | | | 20 | | | 20 | | |
| ALT（IU/L） | 24.74 | ± | 6.41 | 27.84 | ± | 12.71 | 26.30 | ± | 5.34 | 23.09 | ± | 4.38 |
| AST（IU/L） | 88.69 | ± | 19.61 | 102.40 | ± | 45.53 | 90.06 | ± | 20.22 | 77.18 | ± | 19.00 |
| T.BIL（umol/L） | 0.576 | ± | 0.355 | 0.567 | ± | 0.339 | 0.768 | ± | 0.254 | 0.803 | ± | 0.272* |
| ALP（IU/L） | 62.32 | ± | 19.50 | 60.38 | ± | 20.26 | 62.39 | ± | 25.46 | 61.38 | ± | 23.49 |
| γ-GT（IU/L） | 0.60 | ± | 0.25 | 0.65 | ± | 0.19 | 0.68 | ± | 0.18 | 0.70 | ± | 0.24 |
| CK（IU/L） | 275.7 | ± | 126.7 | 278.3 | ± | 111.9 | 229.6 | ± | 92.7 | 218.2 | ± | 118.4 |
| GLU（mmol/L） | 6.731 | ± | 0.706 | 6.280 | ± | 0.663* | 5.955 | ± | 0.547** | 6.097 | ± | 0.778** |
| BUN（umol/L） | 7.256 | ± | 0.989 | 7.477 | ± | 1.512 | 6.533 | ± | 0.786 | 7.039 | ± | 1.200 |
| Crea（umol/L） | 48.86 | ± | 4.98 | 50.21 | ± | 6.54 | 45.61 | ± | 4.06 | 46.35 | ± | 5.13 |
| T.CHO（mmol/L） | 1.643 | ± | 0.265 | 1.733 | ± | 0.234 | 1.776 | ± | 0.363 | 1.781 | ± | 0.411 |
| TG（mmol/L） | 0.655 | ± | 0.240 | 0.746 | ± | 0.299 | 0.855 | ± | 0.492 | 0.593 | ± | 0.193 |
| T.P（g/L） | 61.28 | ± | 4.02 | 62.00 | ± | 4.30 | 61.50 | ± | 5.17 | 60.01 | ± | 3.04 |
| ALB（g/L） | 38.47 | ± | 2.66 | 39.24 | ± | 3.29 | 39.08 | ± | 3.52 | 38.35 | ± | 2.23 |
| GLO (g/L) | 22.81 | ± | 2.57 | 22.77 | ± | 1.87 | 22.42 | ± | 1.99 | 21.65 | ± | 1.86 |
| A/G | 1.70 | ± | 0.19 | 1.73 | ± | 0.16 | 1.75 | ± | 0.11 | 1.78 | ± | 0.16 |
| K^+^（mmol/L） | 3.75 | ± | 0.28 | 3.83 | ± | 0.29 | 3.71 | ± | 0.22 | 3.80 | ± | 0.21 |
| Na^+^（mmol/L） | 138.4 | ± | 1.3 | 139.7 | ± | 1.1** | 140.5 | ± | 0.8** | 140.1 | ± | 1.1** |
| Cl^-^（mmol/L） | 101.9 | ± | 2.5 | 104.0 | ± | 2.3 | 105.7 | ± | 1.8** | 105.8 | ± | 1.8** |
| TCa（mmol/L） | 1.53 | ± | 0.06 | 1.52 | ± | 0.05 | 1.48 | ± | 0.03** | 1.48 | ± | 0.04* |

**Table S4 Effect of aqueous extracts on serum biochemistry in rats at the end of recovery period**

| Test items | Groups | | | | | | | | | | | | |
| --- | --- | --- | --- | --- | --- | --- | --- | --- | --- | --- | --- | --- | --- |
|  | Control | | | Low dose group | | | Middle dose group | | | High dose group | | | |
| Number of rats | 10 | | | 10 | | | 10 | | | 10 | | | |
| ALT（IU/L） | 23.22 | ± | 3.47 | 23.95 | ± | 3.18 | 23.86 | ± | 2.93 | 22.83 | ± | 5.33 |  |
| AST（IU/L） | 90.35 | ± | 20.91 | 75.68 | ± | 11.04 | 84.60 | ± | 16.70 | 93.14 | ± | 15.18 |  |
| T.BIL（umol/L） | 0.704 | ± | 0.435 | 0.713 | ± | 0.284 | 0.411 | ± | 0.346 | 0.474 | ± | 0.326 |  |
| ALP（IU/L） | 55.88 | ± | 18.87 | 59.21 | ± | 26.01 | 52.91 | ± | 20.67 | 55.15 | ± | 22.35 |  |
| γ-GT（IU/L） | 0.48 | ± | 0.25 | 0.37 | ± | 0.30 | 0.37 | ± | 0.17 | 0.46 | ± | 0.28 |  |
| CK（IU/L） | 278.0 | ± | 105.9 | 224.1 | ± | 122.4 | 283.1 | ± | 125.2 | 401.7 | ± | 297.1 |  |
| GLU（mmol/L） | 6.366 | ± | 0.446 | 6.218 | ± | 0.638 | 6.365 | ± | 0.901 | 6.266 | ± | 0.690 |  |
| BUN（umol/L） | 6.826 | ± | 1.260 | 7.581 | ± | 1.771 | 7.867 | ± | 1.575 | 7.728 | ± | 1.294 |  |
| Crea（umol/L） | 58.78 | ± | 8.74 | 59.76 | ± | 8.83 | 59.87 | ± | 8.60 | 56.57 | ± | 7.25 |  |
| T.CHO（mmol/L） | 1.819 | ± | 0.261 | 1.856 | ± | 0.521 | 1.785 | ± | 0.455 | 1.421 | ± | 0.251 |  |
| TG（mmol/L） | 0.727 | ± | 0.220 | 0.792 | ± | 0.241 | 0.671 | ± | 0.177 | 0.605 | ± | 0.165 |  |
| T.P（g/L） | 67.42 | ± | 4.82 | 67.72 | ± | 5.89 | 66.94 | ± | 6.21 | 62.15 | ± | 4.34 |  |
| ALB（g/L） | 39.34 | ± | 3.39 | 39.57 | ± | 4.52 | 39.06 | ± | 4.29 | 36.56 | ± | 2.91 |  |
| GLO (g/L) | 28.08 | ± | 1.67 | 28.14 | ± | 2.04 | 27.89 | ± | 2.13 | 25.59 | ± | 1.55** |  |
| A/G | 1.40 | ± | 0.07 | 1.41 | ± | 0.13 | 1.40 | ± | 0.08 | 1.43 | ± | 0.05 |  |
| K^+^（mmol/L） | 4.04 | ± | 0.37 | 3.80 | ± | 0.20 | 3.87 | ± | 0.14 | 3.90 | ± | 0.27 |  |
| Na^+^（mmol/L） | 138.5 | ± | 0.8 | 138.9 | ± | 0.5 | 139.4 | ± | 1.0 | 138.9 | ± | 0.6 |  |
| Cl^-^（mmol/L） | 108.2 | ± | 1.4 | 108.7 | ± | 1.5 | 108.4 | ± | 1.8 | 109.0 | ± | 1.5 |  |
| TCa（mmol/L） | 1.93 | ± | 0.01 | 1.91 | ± | 0.02** | 1.89 | ± | 0.01** | 1.90 | ± | 0.02** |  |

| **Table S5 Effect of aqueous extracts on serum biochemistry in male rats after drug withdrawal** | | | | | | | | | | | | |  |
| --- | --- | --- | --- | --- | --- | --- | --- | --- | --- | --- | --- | --- | --- |
| Groups | urobilinogen umol/L | bilirubin umol/L | ketone body mmol/L | Blood Ery/ul | Protein g/L | nitrite | hemameba Leuko/ul | glucose mmol/L | Microalbumin g/L | proportion | pH | vitamin C mmol/L | |
|  |  |  |  |  |  |  |  |  |  |  |  |  |  |
| Control | Normal：15/15 | Neg：15/15 | Neg：4/15 | Neg：15/15 | Neg：2/15 | Neg：15/15 | Neg：13/15 | Neg：15/15 | 0.15:2/15 | 1.010±0.004 | 7.37±0.58 | 0.00±0.00 | |
|  |  |  | +-: 10/15  1+: 1/15 |  | Trace: 9/15  1+: 4/15 |  | +-: 2/15 |  | >0.15: 13/15 |  |  |  | |
| Low dose group | Normal：12/14 | Neg：14/14 | Neg：1/14 | Neg：14/14 | Neg：1/14 | Neg：14/14 | Neg：11/14 | Neg：14/14 | 0.15：1/14 | 1.020±0.006 | 7.46±0.46 | 0.20±0.75 | |
|  | 1+: 2/14 |  | +-: 12/14  1+: 1/14 |  | Trace: 8/14  1+: 5/14 |  | +-: 3/14 |  | >0.15: 13/14 |  |  |  | |
| Middle dose group | Normal：15/15 | Neg：15/15 | +-: 12/15  1+: 3/15 | Neg：15/15 | Neg：4/15 | Neg：15/15 | Neg：12/15 | Neg：15/15 | 0.15：4/15 | 1.020±0.004** | 7.07±0.75 | 0.12±0.25 | |
|  |  |  |  |  | Trace: 5/15  1+: 6/15 |  | +-: 2/15  1+: 1/15 |  | >0.15: 11/15 |  |  |  | |
| High dose group | Normal：14/14 | Neg：14/14 | +-: 14/14 | Neg：14/14 | Neg：8/14 | Neg：14/14 | Neg：8/14 | Neg：14/14 | Neg：1/14  0.15：7/14 | 1.030±0.001** | 6.46±0.54** | 0.09±0.22 | |
|  |  |  |  |  | Trace: 6/14 |  | +-: 6/14 |  | >0.15: 6/14* |  |  |  | |
| "Normal" and "Neg" indicate that the indicator is normal, ketone body "+-" indicates that the measured value is 0.5 mmol/L, "1+" indicates that the measured value is 1.5 mmol/L, urobilinogen "1+" indicates that the measured value is 34 μmol / L, protein "trace" indicates that the measured value is trace, "1+" indicates that the measured value is 0.3 g/L, microalbumin "0.15" indicates that the measured value is 0.15 g/L, and ">0.15" indicates that the measured value is >0.15 g/L; hemameba "+-" represents ca.15, "1 +" represents ca.70. | | | | | | | | | | | | |  |

| **Table S6 Effect of aqueous extracts on serum biochemistry in female rats after drug withdrawal** | | | | | | | | | | | | | | | | | |
| --- | --- | --- | --- | --- | --- | --- | --- | --- | --- | --- | --- | --- | --- | --- | --- | --- | --- |
| Groups | urobilinogen umol/L | | bilirubin umol/L | | ketone body mmol/L | Blood Ery/ul | Protein g/L | nitrite | hemameba Leuko/ul | | glucose mmol/L | Microalbumin g/L | | proportion | pH | vitamin C mmol/L |  |
|  |  |  |  |  |  |  |  |  |  |  |  |  |  |  |  |  |  |
| Control | Normal：15/15 | Neg：  15/15 | | Neg：11/15 | | Neg：  15/15 | Neg：  14/15 | Neg：14/15 | Neg：  15/15 | Neg：  15/15 | | Neg：  11/15 | 1.008±0.003 | | 7.03±0.69 | 0.0±0.0 |  |
|  |  |  | | +-：4/15 | |  | Trace：1/15 | Pos：1/15 |  |  | | 0.15：3/15  >0.15:1/15 |  | |  |  |  |
| Low dose group | Normal：15/15 | Neg：15/15 | | Neg：15/15 | | Neg：15/15 | Neg：15/15 | Neg：10/15 | Neg：  15/15 | Neg：15/15 | | Neg：  15/15 | 1.007±0.002 | | 7.23±0.62 | 0.0±0.0 |  |
|  |  |  | |  | |  |  | Pos：5/15 |  |  | |  |  | |  |  |  |
| Middle dose group | Normal：15/15 | Neg：15/15 | | Neg：15/15 | | Neg：15/15 | Neg：15/15 | Neg：  9/15 | Neg：  15/15 | Neg：15/15 | | Neg：  14/15 | 1.009±0.004 | | 7.00±0.57 | 0.0±0.0 |  |
|  |  |  | |  | |  |  | Pos:6/15 |  |  | | 0.15：1/15 |  | |  |  |  |
| High dose group | Normal：15/15 | Neg：15/15 | | Neg：  15/15 | | Neg：15/15 | Neg：  15/15 | Neg：11/15 | Neg：  15/15 | Neg：15/15 | | Neg：  14/15 | 1.008±0.004 | | 7.03±0.72 | 0.0±0.0 |  |
|  |  |  | |  | |  |  | Pos：4/15 |  |  | | 0.15：1/15 |  | |  |  |  |
|  | | | | | | | | | | | | | | | | | |

| **Table S7 Effect of aqueous extracts on serum biochemistry in male rats at the end of recovery period** | | | | | | | | | | | | |  |
| --- | --- | --- | --- | --- | --- | --- | --- | --- | --- | --- | --- | --- | --- |
| Groups | urobilinogen umol/L | bilirubin umol/L | ketone body mmol/L | Blood Ery/ul | Protein g/L | nitrite | hemameba Leuko/ul | glucose mmol/L | Microalbumin g/L | proportion | pH | vitamin C mmol/L | |
|  |  |  |  |  |  |  |  |  |  |  |  |  |  |
| Control | Normal：  5/5 | Neg：  5/5 | Neg：1/5 | Neg：  5/5 | Neg：  4/5 | Neg：4/5 | Neg：  5/5 | Neg:  5/5 | Neg：  2/5  0.15: 2/5 | 1.013±0.003 | 7.30±0.27 | 0±0 | |
|  |  |  | +-: 4/5 |  | Trace:1/5 | Pos:1/5 |  |  | >0.15: 1/5 |  |  |  | |
| Low dose group | Normal：  5/5 | Neg：  5/5 | Neg：4/5  +-: 1/5 | Neg：  5/5 | Neg：  5/5 | Neg：5/5 | Neg：  5/5 | Neg：  5/5 | Neg：  5/5 | 1.013±0.003 | 7.30±0.45 | 0±0 | |
| Middle dose group | Normal：  5/5 | Neg：  5/5 | Neg：4/5  +-: 1/5 | Neg：  5/5 | Neg：  4/5  Trace:1/5 | Neg：5/5 | Neg：  5/5 | Neg：  5/5 | Neg：  3/5  0.15:1/5 | 1.010±0.000 | 7.60±0.42 | 0±0 | |
|  |  |  |  |  |  |  |  |  | >0.15:1/5 |  |  |  | |
| High dose group | Normal：  5/5 | Neg：  5/5 | Neg：5/5 | Neg：  5/5 | Neg：  5/5 | Neg：5/5 | Neg：  5/5 | Neg：  5/5 | Neg：  4/5 | 1.014±0.004 | 7.60±0.22 | 0±0 | |
|  |  |  |  |  |  |  |  |  | 0.15:1/5 |  |  |  | |
|  | | | | | | | | | | | | |  |

| **Table S8 Effect of aqueous extracts on serum biochemistry in female rats at the end of recovery period** | | | | | | | | | | | | |  |
| --- | --- | --- | --- | --- | --- | --- | --- | --- | --- | --- | --- | --- | --- |
| Groups | urobilinogen umol/L | bilirubin umol/L | ketone body mmol/L | Blood Ery/ul | Protein g/L | nitrite | hemameba Leuko/ul | glucose mmol/L | Microalbumin g/L | proportion | pH | vitamin C mmol/L | |
|  |  |  |  |  |  |  |  |  |  |  |  |  |  |
| Control | Normal：  5/5 | Neg：  5/5 | Neg：  4/5 | Neg：  5/5 | Neg：  5/5 | Neg：  5/5 | Neg：  5/5 | Neg：  5/5 | Neg：  5/5 | 0.811±0.453 | 7.20±0.27 | 0±0 | |
|  |  |  | +-: 1/5 |  |  |  |  |  |  |  |  |  | |
| Low dose group | Normal：  5/5 | Neg：  5/5 | Neg：  2/5  　+-: 3/5 | Neg：  5/5 | Neg：  5/5 | Neg：  5/5 | Neg：  5/5 | Neg：  5/5 | Neg：  4/5  0.15: 1/5 | 1.012±0.003 | 7.50±0.00 | 0±0 | |
| Middle dose group | Normal：  5/5 | Neg：  5/5 | Neg：  2/5 | Neg：  5/5 | Neg：  3/5 | Neg：  5/5 | Neg：  5/5 | Neg：  5/5 | Neg：  2/5  0.15: 1/5 | 1.014±0.005 | 7.50±0.35 | 0±0 | |
|  |  |  | +-: 3/5 |  | Trace:2/5 |  |  |  | >0.15:2/5 |  |  |  | |
| High dose group | Normal：  5/5 | Neg：  5/5 | +-:  5/5 | Neg：  4/5  +-:1/5 | Neg：  5/5 | Neg：  5/5 | Neg：  5/5 | Neg：  5/5 | 0.15:  5/5** | 1.012±0.003 | 6.90±0.82 | 0±0 | |
|  |  |  |  |  |  |  |  |  |  |  |  |  | |
| \|  \| \| --- \| | | | | | | | | | | | | |  |

**Table S9 Effect of aqueous extracts on ophthalmic indexes of rats**

| Groups | examination phase | Number of rats | Eye appearance | | | | | | | | Anterior segment | | | | | | | | | Posterior segment | | | | | | |
| --- | --- | --- | --- | --- | --- | --- | --- | --- | --- | --- | --- | --- | --- | --- | --- | --- | --- | --- | --- | --- | --- | --- | --- | --- | --- | --- |
|  |  |  | Eyelid | Orbit | Eyeball | | | Conjun-ctiva | Lacrimal apparatus | | Cornea | | Sclera | anterior chamber | | | Iris | Pupil | | Lens | | Vitreous | Papilla of optic nerve | | Retina | |
| Control | Withdrawal examination | 30 | Neg：30/30 | Neg：30/30 | | Neg：30/30 | Neg：30/30 | | Neg：30/30 | Neg：30/30 | | Neg：30/30 | | | Neg：30/30 | Neg：30/30 | | | Neg：30/30 | | Neg：30/30 | Neg：  30/30 | | Neg：30/30 | | Neg：30/30 |
|  | Examination at the end of recovery period | 10 | Neg：  10/10 | Neg：  10/10 | | Neg：  10/10 | Neg：  10/10 | | Neg：  10/10 | Neg：  10/10 | | Neg：  10/10 | | | Neg：  10/10 | Neg：  10/10 | | | Neg：  10/10 | | Neg：  10/10 | Neg：  10/10 | | Neg：  10/10 | | Neg：  10/10 |
| Low dose group | Withdrawal examination | 30 | Neg：30/30 | Neg：30/30 | | Neg：30/30 | Neg：30/30 | | Neg：30/30 | Neg：30/30 | | Neg：30/30 | | | Neg：30/30 | Neg：30/30 | | | Neg：30/30 | | Neg：30/30 | Neg：  30/30 | | Neg：30/30 | | Neg：30/30 |
|  | Examination at the end of recovery period | 10 | Neg：  10/10 | Neg：  10/10 | | Neg：  10/10 | Neg：  10/10 | | Neg：  10/10 | Neg：  10/10 | | Neg：  10/10 | | | Neg：  10/10 | Neg：  10/10 | | | Neg：  10/10 | | Neg：  10/10 | Neg：  10/10 | | Neg：  10/10 | | Neg：  10/10 |
| Middle dose group | Withdrawal examination | 30 | Neg：30/30 | Neg：30/30 | | Neg：30/30 | Neg：30/30 | | Neg：30/30 | Neg：30/30 | | Neg：30/30 | | | Neg：30/30 | Neg：30/30 | | | Neg：30/30 | | Neg：30/30 | Neg：  30/30 | | Neg：30/30 | | Neg：30/30 |
|  | Examination at the end of recovery period | 10 | Neg：  10/10 | Neg：  10/10 | | Neg：  10/10 | Neg：  10/10 | | Neg：  10/10 | Neg：  10/10 | | Neg：  10/10 | | | Neg：  10/10 | Neg：  10/10 | | | Neg：  10/10 | | Neg：  10/10 | Neg：  10/10 | | Neg：  10/10 | | Neg：  10/10 |
| High dose group | Withdrawal examination | 30 | Neg：30/30 | Neg：30/30 | | Neg：30/30 | Neg：30/30 | | Neg：30/30 | Neg：30/30 | | Neg：30/30 | | | Neg：30/30 | Neg：30/30 | | | Neg：30/30 | | Neg：30/30 | Neg：  30/30 | | Neg：30/30 | | Neg：30/30 |
|  | Examination at the end of recovery period | 10 | Neg：  10/10 | Neg：  10/10 | | Neg：  10/10 | Neg：  10/10 | | Neg：  10/10 | Neg：  10/10 | | Neg：  10/10 | | | Neg：  10/10 | Neg：  10/10 | | | Neg：  10/10 | | Neg：  10/10 | Neg：  10/10 | | Neg：  10/10 | | Neg：  10/10 |

"Neg" indicates that the index is normal.

**Table S10 Effect of aqueous extracts on organ weight (g) and organ** **coefficient (%) in male rats after drug withdrawal**

| Test items | Groups | | | | | | | | | | | |
| --- | --- | --- | --- | --- | --- | --- | --- | --- | --- | --- | --- | --- |
|  | Control | | | Low dose group | | | Middle dose group | | | High dose group | | |
| Number of rats | 10 | | | 10 | | | 10 | | | 10 | | |
| Weight（g） | 523.1 | ± | 35.4 | 492.2 | ± | 33.0 | 489.4 | ± | 60.5 | 469.7 | ± | 35.0 |
| Heart（g） | 1.634 | ± | 0.106 | 1.535 | ± | 0.143 | 1.503 | ± | 0.186 | 1.471 | ± | 0.097 |
| Heart（%） | 0.313 | ± | 0.023 | 0.312 | ± | 0.023 | 0.308 | ± | 0.020 | 0.314 | ± | 0.012 |
| Liver（g） | 15.415 | ± | 1.811 | 13.668 | ± | 1.642* | 13.394 | ± | 2.275* | 13.226 | ± | 1.526* |
| Liver（%） | 2.940 | ± | 0.186 | 2.772 | ± | 0.204 | 2.724 | ± | 0.172 | 2.812 | ± | 0.194 |
| Spleen（g） | 0.983 | ± | 0.128 | 0.918 | ± | 0.143 | 0.965 | ± | 0.174 | 0.901 | ± | 0.140 |
| Spleen（%） | 0.188 | ± | 0.023 | 0.186 | ± | 0.022 | 0.200 | ± | 0.046 | 0.191 | ± | 0.020 |
| Kidney（g） | 3.748 | ± | 0.309 | 3.527 | ± | 0.335 | 3.552 | ± | 0.417 | 3.716 | ± | 0.354 |
| Kidney（%） | 0.718 | ± | 0.050 | 0.718 | ± | 0.073 | 0.729 | ± | 0.063 | 0.792 | ± | 0.063* |
| Brain（g） | 2.271 | ± | 0.089 | 2.294 | ± | 0.063 | 2.287 | ± | 0.097 | 2.241 | ± | 0.132 |
| Brain（%） | 0.436 | ± | 0.031 | 0.468 | ± | 0.036 | 0.473 | ± | 0.051 | 0.479 | ± | 0.035 |
| Thymus（g） | 0.334 | ± | 0.051 | 0.370 | ± | 0.105 | 0.270 | ± | 0.056 | 0.294 | ± | 0.080 |
| Thymus（%） | 0.064 | ± | 0.010 | 0.076 | ± | 0.021 | 0.055 | ± | 0.010 | 0.062 | ± | 0.015 |
| Paranephros（g） | 0.069 | ± | 0.008 | 0.066 | ± | 0.011 | 0.066 | ± | 0.014 | 0.066 | ± | 0.011 |
| Paranephros（%） | 0.013 | ± | 0.002 | 0.014 | ± | 0.003 | 0.014 | ± | 0.003 | 0.014 | ± | 0.003 |
| Testis（g） | 4.035 | ± | 0.298 | 4.054 | ± | 0.197 | 3.970 | ± | 0.301 | 3.960 | ± | 0.225 |
| Testis（%） | 0.776 | ± | 0.093 | 0.826 | ± | 0.048 | 0.820 | ± | 0.099 | 0.847 | ± | 0.081 |
| Epididymis（g） | 1.666 | ± | 0.184 | 1.606 | ± | 0.156 | 1.571 | ± | 0.182 | 1.622 | ± | 0.131 |
| Epididymis（%） | 0.320 | ± | 0.040 | 0.327 | ± | 0.026 | 0.324 | ± | 0.042 | 0.347 | ± | 0.032 |
| Prostate（g） | 3.512 | ± | 0.334 | 3.365 | ± | 0.693 | 3.498 | ± | 0.737 | 3.113 | ± | 0.415 |
| Prostate（%） | 0.673 | ± | 0.062 | 0.688 | ± | 0.160 | 0.717 | ± | 0.138 | 0.670 | ± | 0.130 |

**Table S11 Effect of aqueous extract on organs weight (g) and organ** **coefficient (%) in female rats after drug withdrawal**

| Test items | Groups | | | | | | | | | | | |
| --- | --- | --- | --- | --- | --- | --- | --- | --- | --- | --- | --- | --- |
|  | Control | | | Low dose group | | | Middle dose group | | | High dose group | | |
| Number of rats | 10 | | | 10 | | | 10 | | | 10 | | |
| Weight（g） | 276.3 | ± | 15.7 | 272.0 | ± | 20.7 | 268.4 | ± | 11.1 | 277.2 | ± | 24.2 |
| Heart（g） | 0.932 | ± | 0.050 | 0.933 | ± | 0.065 | 0.981 | ± | 0.058 | 0.959 | ± | 0.075 |
| Heart（%） | 0.338 | ± | 0.014 | 0.345 | ± | 0.035 | 0.366 | ± | 0.017 | 0.347 | ± | 0.027 |
| Liver（g） | 7.658 | ± | 0.799 | 7.858 | ± | 0.650 | 7.823 | ± | 0.680 | 7.876 | ± | 0.514 |
| Liver（%） | 2.770 | ± | 0.217 | 2.904 | ± | 0.324 | 2.916 | ± | 0.244 | 2.849 | ± | 0.155 |
| Spleen（g） | 0.655 | ± | 0.077 | 0.654 | ± | 0.124 | 0.616 | ± | 0.088 | 0.629 | ± | 0.081 |
| Spleen（%） | 0.238 | ± | 0.031 | 0.240 | ± | 0.040 | 0.230 | ± | 0.031 | 0.227 | ± | 0.021 |
| Kidney（g） | 2.034 | ± | 0.174 | 1.980 | ± | 0.171 | 2.081 | ± | 0.127 | 2.112 | ± | 0.159 |
| Kidney（%） | 0.736 | ± | 0.045 | 0.733 | ± | 0.099 | 0.776 | ± | 0.047 | 0.765 | ± | 0.067 |
| Brain（g） | 2.104 | ± | 0.075 | 2.104 | ± | 0.086 | 2.101 | ± | 0.094 | 2.090 | ± | 0.099 |
| Brain（%） | 0.764 | ± | 0.046 | 0.778 | ± | 0.068 | 0.784 | ± | 0.049 | 0.757 | ± | 0.048 |
| Thymus（g） | 0.264 | ± | 0.061 | 0.221 | ± | 0.050 | 0.246 | ± | 0.062 | 0.246 | ± | 0.072 |
| Thymus（%） | 0.096 | ± | 0.020 | 0.082 | ± | 0.022 | 0.092 | ± | 0.024 | 0.088 | ± | 0.022 |
| Paranephros（g） | 0.077 | ± | 0.013 | 0.071 | ± | 0.010 | 0.075 | ± | 0.013 | 0.072 | ± | 0.013 |
| Paranephros（%） | 0.028 | ± | 0.005 | 0.026 | ± | 0.004 | 0.028 | ± | 0.004 | 0.026 | ± | 0.005 |
| Uterus（g） | 0.899 | ± | 0.202 | 0.927 | ± | 0.431 | 0.859 | ± | 0.244 | 0.854 | ± | 0.356 |
| Uterus（%） | 0.327 | ± | 0.080 | 0.343 | ± | 0.171 | 0.320 | ± | 0.087 | 0.314 | ± | 0.136 |
| Ovary（g） | 0.108 | ± | 0.025 | 0.112 | ± | 0.037 | 0.098 | ± | 0.019 | 0.105 | ± | 0.043 |
| Ovary（%） | 0.039 | ± | 0.009 | 0.041 | ± | 0.013 | 0.036 | ± | 0.007 | 0.039 | ± | 0.016 |

**Table S12 Effect of aqueous extracts on organ weight (g) and organ** **coefficient (%) in male rats at the end of recovery period**

| Test items | Groups | | | | | | | | | | | |
| --- | --- | --- | --- | --- | --- | --- | --- | --- | --- | --- | --- | --- |
|  | Control | | | Low dose group | | | Middle dose group | | | High dose group | | |
| Number of rats | 5 | | | 5 | | | 5 | | | 5 | | |
| Weight（g） | 498.8 | ± | 11.5 | 546.8 | ± | 46.4 | 532.6 | ± | 63.4 | 512.0 | ± | 29.0 |
| Heart（g） | 1.487 | ± | 0.147 | 1.572 | ± | 0.144 | 1.642 | ± | 0.207 | 1.441 | ± | 0.168 |
| Heart（%） | 0.299 | ± | 0.035 | 0.288 | ± | 0.014 | 0.309 | ± | 0.020 | 0.281 | ± | 0.024 |
| Liver（g） | 12.888 | ± | 0.964 | 14.403 | ± | 1.986 | 13.599 | ± | 2.179 | 13.467 | ± | 1.139 |
| Liver（%） | 2.583 | ± | 0.171 | 2.625 | ± | 0.146 | 2.545 | ± | 0.119 | 2.630 | ± | 0.157 |
| Spleen（g） | 0.996 | ± | 0.145 | 0.986 | ± | 0.105 | 0.954 | ± | 0.081 | 0.882 | ± | 0.118 |
| Spleen（%） | 0.199 | ± | 0.027 | 0.181 | ± | 0.022 | 0.180 | ± | 0.008 | 0.171 | ± | 0.014 |
| Kidney（g） | 3.481 | ± | 0.377 | 3.597 | ± | 0.284 | 3.752 | ± | 0.418 | 3.611 | ± | 0.352 |
| Kidney（%） | 0.698 | ± | 0.075 | 0.660 | ± | 0.059 | 0.707 | ± | 0.059 | 0.705 | ± | 0.039 |
| Brain（g） | 2.287 | ± | 0.141 | 2.378 | ± | 0.080 | 2.321 | ± | 0.036 | 2.271 | ± | 0.105 |
| Brain（%） | 0.458 | ± | 0.023 | 0.438 | ± | 0.041 | 0.440 | ± | 0.046 | 0.444 | ± | 0.030 |
| Thymus（g） | 0.255 | ± | 0.104 | 0.229 | ± | 0.083 | 0.228 | ± | 0.067 | 0.288 | ± | 0.065 |
| Thymus（%） | 0.051 | ± | 0.020 | 0.041 | ± | 0.012 | 0.043 | ± | 0.014 | 0.056 | ± | 0.011 |
| Paranephros（g） | 0.055 | ± | 0.005 | 0.070 | ± | 0.008** | 0.062 | ± | 0.009 | 0.060 | ± | 0.006 |
| Paranephros（%） | 0.011 | ± | 0.001 | 0.013 | ± | 0.002 | 0.012 | ± | 0.003 | 0.012 | ± | 0.002 |
| Testis（g） | 3.829 | ± | 0.235 | 4.054 | ± | 0.110 | 4.170 | ± | 0.240 | 3.953 | ± | 0.437 |
| Testis（%） | 0.761 | ± | 0.041 | 0.747 | ± | 0.080 | 0.789 | ± | 0.071 | 0.772 | ± | 0.062 |
| Epididymis（g） | 1.543 | ± | 0.068 | 1.607 | ± | 0.098 | 1.554 | ± | 0.088 | 1.557 | ± | 0.192 |
| Epididymis（%） | 0.307 | ± | 0.010 | 0.295 | ± | 0.026 | 0.295 | ± | 0.033 | 0.304 | ± | 0.035 |
| Prostate（g） | 3.603 | ± | 0.185 | 4.385 | ± | 0.414 | 4.214 | ± | 0.583 | 3.969 | ± | 0.622 |
| Prostate（%） | 0.723 | ± | 0.048 | 0.806 | ± | 0.102 | 0.799 | ± | 0.141 | 0.776 | ± | 0.113 |

**Table S13 Effect of aqueous extracts on organ weight (g) and organ** **coefficient (%) in female rats at the end of recovery period**

| Test items | Groups | | | | | | | | | | | |
| --- | --- | --- | --- | --- | --- | --- | --- | --- | --- | --- | --- | --- |
|  | Control | | | Low dose group | | | Middle dose group | | | High dose group | | |
| Number of rats | 5 | | | 5 | | | 5 | | | 5 | | |
| Weight（g） | 284.4 | ± | 21.3 | 273.2 | ± | 19.1 | 281.2 | ± | 13.8 | 276.6 | ± | 21.7 |
| Heart（g） | 0.969 | ± | 0.095 | 0.919 | ± | 0.095 | 0.951 | ± | 0.085 | 0.871 | ± | 0.085 |
| Heart（%） | 0.340 | ± | 0.014 | 0.336 | ± | 0.021 | 0.338 | ± | 0.021 | 0.315 | ± | 0.019 |
| Liver（g） | 7.935 | ± | 0.497 | 7.648 | ± | 0.800 | 7.698 | ± | 0.509 | 7.748 | ± | 0.902 |
| Liver（%） | 2.794 | ± | 0.125 | 2.797 | ± | 0.191 | 2.737 | ± | 0.091 | 2.795 | ± | 0.112 |
| Spleen（g） | 0.617 | ± | 0.058 | 0.593 | ± | 0.049 | 0.587 | ± | 0.041 | 0.591 | ± | 0.090 |
| Spleen（%） | 0.218 | ± | 0.020 | 0.217 | ± | 0.012 | 0.209 | ± | 0.012 | 0.214 | ± | 0.034 |
| Kidney（g） | 1.942 | ± | 0.134 | 1.864 | ± | 0.157 | 1.892 | ± | 0.091 | 1.984 | ± | 0.154 |
| Kidney（%） | 0.684 | ± | 0.035 | 0.683 | ± | 0.053 | 0.674 | ± | 0.041 | 0.718 | ± | 0.026 |
| Brain（g） | 2.200 | ± | 0.175 | 2.149 | ± | 0.073 | 2.161 | ± | 0.142 | 2.091 | ± | 0.136 |
| Brain（%） | 0.775 | ± | 0.056 | 0.790 | ± | 0.058 | 0.770 | ± | 0.059 | 0.757 | ± | 0.042 |
| Thymus（g） | 0.164 | ± | 0.032 | 0.191 | ± | 0.058 | 0.175 | ± | 0.016 | 0.212 | ± | 0.038 |
| Thymus（%） | 0.058 | ± | 0.012 | 0.070 | ± | 0.018 | 0.062 | ± | 0.005 | 0.077 | ± | 0.017 |
| Paranephros（g） | 0.066 | ± | 0.014 | 0.065 | ± | 0.012 | 0.068 | ± | 0.007 | 0.075 | ± | 0.015 |
| Paranephros（%） | 0.023 | ± | 0.004 | 0.024 | ± | 0.004 | 0.024 | ± | 0.003 | 0.027 | ± | 0.006 |
| Uterus（g） | 0.975 | ± | 0.584 | 0.876 | ± | 0.352 | 0.826 | ± | 0.257 | 0.991 | ± | 0.326 |
| Uterus（%） | 0.349 | ± | 0.219 | 0.319 | ± | 0.117 | 0.294 | ± | 0.091 | 0.359 | ± | 0.117 |
| Ovary（g） | 0.116 | ± | 0.005 | 0.109 | ± | 0.021 | 0.092 | ± | 0.009 | 0.110 | ± | 0.012 |
| Ovary（%） | 0.041 | ± | 0.004 | 0.040 | ± | 0.010 | 0.033 | ± | 0.003 | 0.040 | ± | 0.002 |

Figure S1 MS information of the major compounds

Peak 1

**
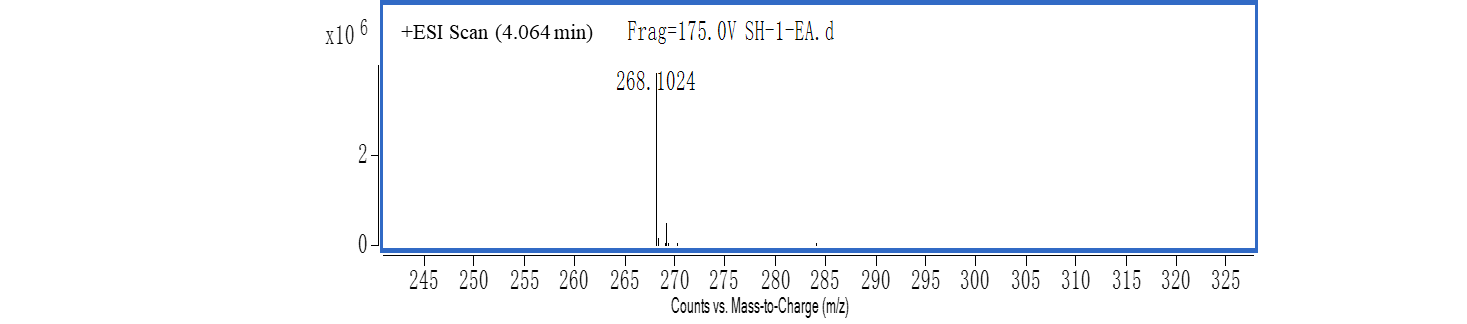
**

Peak 2

**
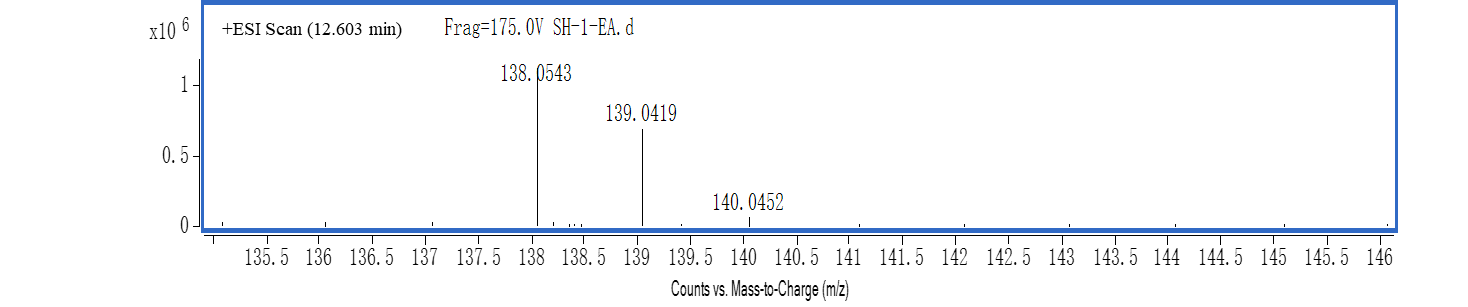
**

Peak 3

Peak 4

Peak 5
